# Mixed features in depression: frequency and associated factors

**DOI:** 10.1192/j.eurpsy.2022.1053

**Published:** 2022-09-01

**Authors:** O. Charaa, U. Ouali, Y. Zgueb, A. Aissa, R. Jomli, F. Nacef

**Affiliations:** Razi hospital, Psychiatry A Department, manouba, Tunisia

**Keywords:** Mixed features, Depression

## Abstract

**Introduction:**

Mixed states in mood disorders present significant clinical and prognostic challenges. Although the DSM-5 has broadened diagnostic criteria for mixed states with the development of the ‘mixed features’ specifier and its application to unipolar depressive disorders, some mixed episodes might still be overlooked.

**Objectives:**

to evaluate the frequency and the factors associated with mixed depression according to the broader Koukopoulos criteria in a sample of patients with a major depressive episode

**Methods:**

We included 99 consecutive patients presenting for a major depressive episode of bipolar (n=10) or unipolar major depressive (n=89) disorder at our outpatient clinic. Major depression was ascertained using SCID- IV criteria, and mixed features were determined using Koukopoulos’ diagnostic criteria

**Results:**

Mean age of the sample was 35.5 years [14-58]. Women accounted for 63.6% of patients. Mixed features were found in 19.5% (n=19) of the sample, 80% (n=8) among patients with bipolar disorders (BD) and 12.3% (n=11) among those with major depressive disorder (MDD). Individuals with mixed features had more substance abuse (p=0.005) and more suicide attempts (p=0.01). Individuals receiving antipsychotics had a lower risk of mixed features (p=0.000) while antidepressant treatment did not have any affect. A family history of BD, psychosis, suicide and substance abuse were found in these patients. Mixed features in depression were more frequent in patients with BD than in MDD.

**Conclusions:**

Our study showed a high frequency of mixed features in depression, especially bipolar depression when Koukopoulos criteria are applied. Special attention should be given to these patients given the association with substance use and suicidality

**Disclosure:**

No significant relationships.

